# Application of intraoperative limb-length measurement by a new osteotomy device in hemiarthroplasty for treating femoral neck fracture

**DOI:** 10.1186/s12893-017-0256-4

**Published:** 2017-05-15

**Authors:** Zhanle Zheng, Lei Yang, Yanling Su, Xian Yu, Zhiyong Hou, Yingze Zhang

**Affiliations:** 1 Department of Orthopedic, The Third Hospital of Hebei Medical University and the Key Biomechanical Laboratory of Orthopaedics, No.139, Ziqiang Road, Shijiazhuang, Hebei province 050051 China; 2 Department of endocrinology, The General Hospital of Hebei Province, Hebei Medical University, Taihua Street, Shijiazhuang, China

**Keywords:** Hemiarthroplasty, Femoral neck fracture, Limb length discrepancy, Measurement, Osteotomy

## Abstract

**Background:**

Limb length discrepancy is one of the most common complications after hip arthroplasty. We developed a device - intraoperative limb-length measurement and osteotomy device (ILMOD), and applied it to patients who were treated with hemiarthroplasty for femoral neck fracture to improve limb length discrepancy by providing an accurate osteotomy during hemi-arthroplasty.

**Methods:**

Between April 2012 and October 2013, 65 patients were treated with hip hemiarthroplasty for femoral neck fracture at our trauma center. 31 patients met the inclusion criteria and were randomly enrolled into two groups ILMOD group and control group. Hemiarthroplasty in this study was performed with cement fixation. Treatment-related measurements such as the operation time, attempts of osteotomy, and the volume of intra-operative blood loss were collected. In both groups, postoperative (1 month) radiologic analysis on anteroposterior weight-bearing pelvic view was performed to evaluate limb length discrepancy.

**Results:**

The results showed significant improvement in limb length discrepancy in ILMOD group, and analysis of postoperative radiographs found the mean length difference is 2.1 ± 1.9 mm in ILMOD group compared to 8.8 ± 5.1 mm in control group (*P <* 0.0001). No complications associated with the use of the device were reported, and none of the patients complained of the discomfort related to limb-length discrepancy after surgery. The average intra-operative time was significantly longer in ILMOD group (84.9 ± 9.2 min) compared to that in control group (70.9 ± 10.2 min) (*P =* 0.0004).

**Conclusions:**

The ILMOD is an effective device that can be used easily for intraoperative limb length measurement and osteotomy during hemiarthroplasty. This method is applicable with Kocher-Langenbeck approach, and the technique could also be used in total hip arthroplasty.

**Trial registration:**

Chinese Clinical Trial Registry ChiCTR-OOC-15005904. Registered 30 Junuary 2015.

## Introduction

Hemiarthroplasty is the treatment choice for femoral neck fractures in elderly patients. Limb-length discrepancy (LLD) is a common complaint after hip arthroplasty and can be a cause of patient dissatisfaction and litigation [1, 2]. Williamson [3] reported that 27% of patients required heel lifts after hip arthroplasty to improve quality of gait. Many methods of measuring limb length directly or indirectly during hip arthroplasty have been reported [4–6].

The incorrect height or angle of osteotomy is one of the most important reasons of LLD, especially for the collared stem. However, most reports did not even take the height and angle of osteotomy into consideration. In efforts to improve LLD, we developed a new device, named intraoperative limb-length measurement and osteotomy device (ILMOD), which can not only measure the height but also optimize the height and angle of osteotomy.

Our study was designed to assess the reliability of the ILMOD during hemiarthroplasty to control limb length and osteotomy perioperatively. The hypothesis was that the ILMOD would improve LLD through a better control of lower-limb length and osteotomy.

## Materials and methods

### Patients

Between April 2012 and October 2013, all patients treated at our trauma center with hemiarthroplasty for femoral neck fractures were enrolled in our study. Inclusion criteria included patients older than 75 years old and those who can walk without aids and lameness before injury. Exclusion criteria were patients younger than 75 years old, with pathologic fractures or patients suffering from serious systemic diseases and could not tolerate the operation. Out of 65 consecutive patients, there were 31 patients who met the inclusion criteria. These were randomly enrolled into two groups: 16 patients in ILMOD group and 15 patients in control group. All hemiarthroplasties in this study were performed with cement fixation and the classic plus hip stems and vario-cups (LINK, Germany) were used. This study was approved by the Ethics Committee of the Third Hospital of Hebei Medical University and all participants gave signed informed consent.

### Introduction of ILMOD

**Fig. 1:**
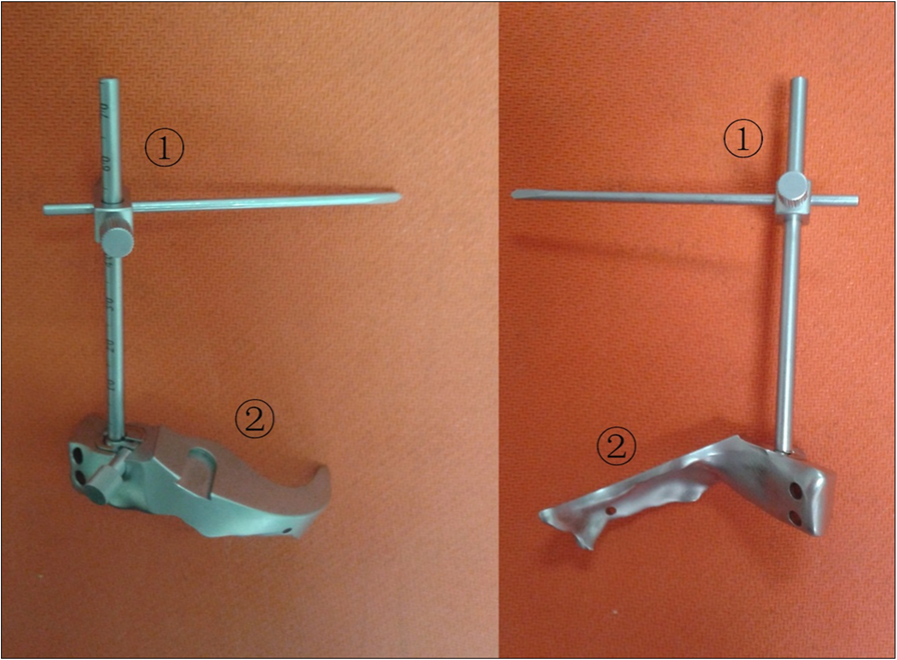
The ILMOD is made of two main parts –a measuring ruler arm ① and a femoral osteotomy guide ②

**Fig. 2:**
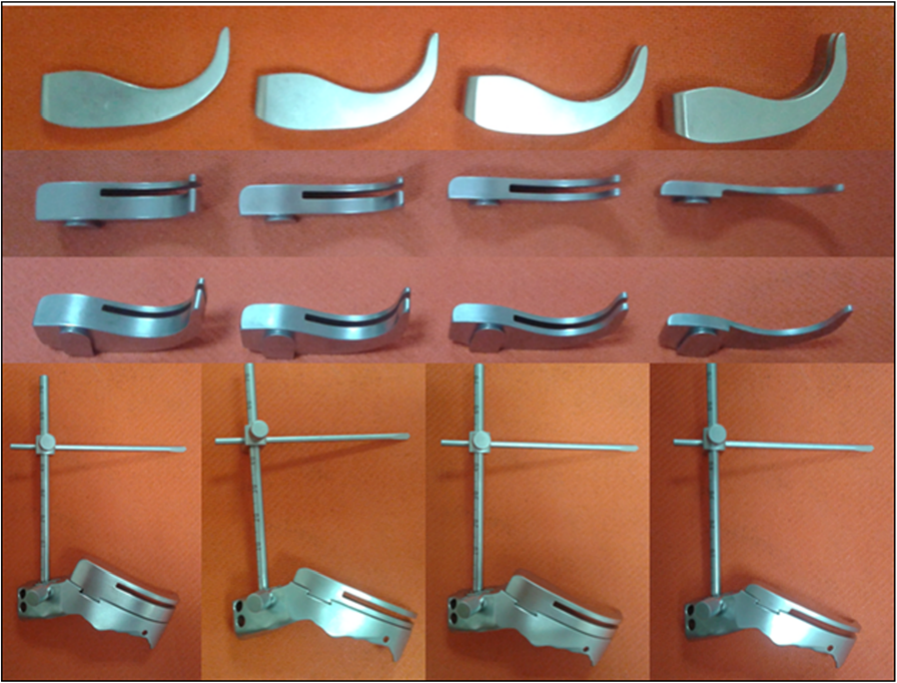
The osteotomy guide can change osteotomy height with the help of clouts, which come with different heights

The ILMOD is made of two main parts - a femoral osteotomy guide and a measuring ruler arm (Fig. 1). The osteotomy guide can be referenced against on the lesser trochanter and against and conforms to landmarks on the body of the femur neck between the femoral head and lesser trochanter. The osteotomy guide was additionally fixed with temporary pins through the guide into greater trochanter. The osteotomy guide has a “C” sharp cutting guide surface to guide a reciprocating saw along cutting surface to create a flat resection on the femoral neck. Furthermore, the osteotomy guide can change osteotomy height with the help of clouts, which come with different heights (Fig. 2). The measuring ruler arm is used to measure the limp length during operation. The distal end of the ruler connect with the osteotomy guide. The proximal end of the measuring ruler arm fits over the guide pin via a protractor guide with locking screw.

Compare with our former femoral neck osteotomy guide device [7], the new intraoperative limb-length measurement and osteotomy device (ILMOD) has five new advances over our previous study. ① The ILMOD adds a measuring ruler arm which can further dismiss the limb length discrepancy. The measuring ruler can be used to aid the proper placement of the femoral component. ② The ILMOD can be fixed on greater trochanter and do not need to be removed during operation which can control the bias caused by repeating mount. ③The ILMOD adds the clouts which come with different heights and can help osteotomy guide changing osteotomy height. The affected limb length cannot be measured when the patient is suffered femoral neck fracture, but this device can measure the length of the femoral neck during operation. ④The most important difference is that the former femoral neck osteotomy guide device emphasis on osteotomy so that easily cause limb length discrepancy, but our new ILMOD emphasis on limb-length measurement during operation which can control limb length better.

### Surgical technique

Before operation, according to the anteroposterior radiograph of the pelvis with bilateral hips in neutral position, the templates were used to determine the angle and height of the femoral neck osteotomy and the potential correct sizes for the femoral components of the prostheses. And a proper ILMOD with/without a clout was chosen and sterilized.

Patients in ILMOD group were operated on using a Kocher-Langenbeck approach, in lateral decubitus. After fracture site was exposed, the femoral head was taken out, and then an anatomical reduction of femoral neck fracture was achieved and fixed by K-wire temporarily (Fig. 3①). The ILMOD was seated on the lesser trochanter and fixed with the preoperative designed height and direction. The guide pin was pressed on the highest point of the femoral head and the measurement value on the ruler arm was recorded (Fig. 3②). Then the K-wire was removed and a reciprocating saw was guided by cutting surface of osteotomy guide to create a flat resection on the femoral neck (Fig. 3③). After reduction with the trial components in place, the ILMOD was replaced and the guide pin was pressed on the highest point of the trial component (Fig. 3④). Intraoperative limb-length discrepancy can be assessed by measuring the change in distance and the desired length achieved by varying neck length of the femoral prosthesis. Once the same height was obtained, a femoral prosthesis (LINK, Germany) which included appropriate leg length dimensions was inserted into the prepared femur at an appropriate angle of anteversion.

**Fig. 3:**
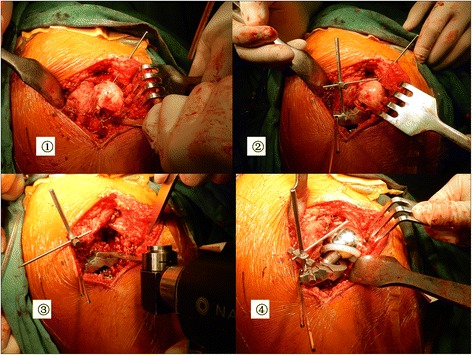
①The femoral head was taken out, and then an anatomical reduction of femoral neck fracture was achieved and fixed by K-wire temporarily; ② The ILMOD was seated on the lesser trochanter. The guide pin was pressed on the highest point of the femoral head; ③ A reciprocating saw was guided by cutting surface of osteotomy guide to create a flat resection on the femoral neck; ④ The guide pin was pressed on the highest point of the trial component

Patients in control group were performed hemiarthroplasty using a Kocher-Langenbeck approach and peroperative stability tests were systematically made to determine femoral prosthesis type.

### Data collection

Patients’ demographic details at presentation were recorded. Treatment-related measurements such as operation time, attempts of osteotomy and volume of intraoperative blood loss were collected.

**Fig. 4:**
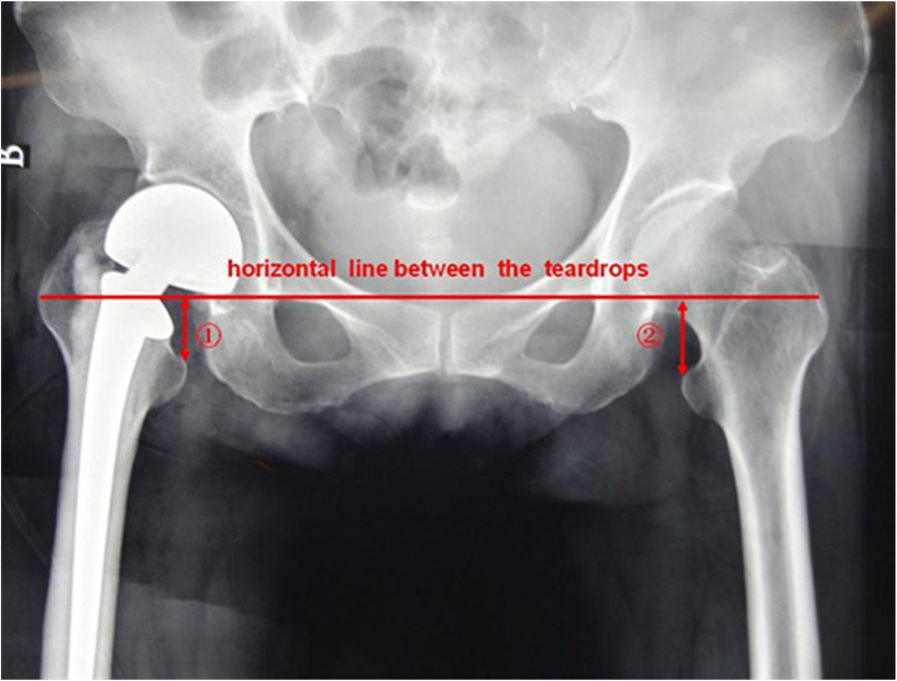
One month postoperative radiologic analysis was performed on anteroposterior weight-bearing pelvic view with bilateral hips, measuring bilateral heights between the horizontal line between the teardrops and the most medial point of the lesser trochanter

In both groups, the follow-up period was 1 month, and major complications (infection, fracture, dislocation, subsidence) were recorded. One month postoperative radiologic analysis was performed on anteroposterior weight-bearing pelvic view with bilateral hips in neutral position, measuring bilateral heights between the horizontal line between the teardrops and the most medial point of the lesser trochanter, to evaluate limb-length discrepancy (Fig. 4). To avoid bias related to the radiograph scale, the long ruler was in the same frontal plane of femur when X-ray image was taken. All differential measurements were recorded as absolute values.

### Statistics

All calculations were made using SPSS18.0 software (SPSS Company, USA). Basic descriptive statistical analyses were used to describe the patient population and treatment outcomes. Statistical analyses were performed using the Student’s t-test or rank sum test. The significance threshold was set at *P* ≤ 0.05. Values were reported as mean ± standard deviation of the mean.

## Results

The mean patient ages at the time of surgery were 80.7 years in ILMOD group and 81.3 years in control group. The LLD showed significant improvement in ILMOD group, and analysis of postoperative radiographs found the mean length difference is 2.1 ± 1.9 mm with the ILMOD and 8.8 ± 5.1 mm without ILMOD (*P <* 0.0001).

No complications associated with the use of the ILMOD occurred, and none of the patients expressed dissatisfaction related to limb-length discrepancy after surgery. There were no other major complications reported postoperatively and 1 month later, such as dislocation, infection, fracture, or sciatic nerve palsy in this series. There was no case of femoral stem subsidence on 1-month postoperative radiographs.

The average intra-operative time was significantly longer in ILMOD group than in control group (84.9 ± 9.2 min vs 70.9 ± 10.2 min, *P =* 0.0004). During operation, all patients in ILMOD group needed only cut the bone once with no need of additional osteotomy. On the other hand, 6 patients in control group needed osteotomies-twice in order to optimize the limb length equality. The intra-operative blood loss averaged 145.9 ± 46.7 ml in the ILMOD group and 125.3 ± 39.4 ml in the control group, with no statistically significant difference (Table 1).

**Table 1:** Demographic and result characteristics

Parameter	ILMOD (*n =* 16)	Control (*n =* 15)	*p-*value
Age (year)	80.7 ± 3.7 (range, 75–87)	81.3 ± 4.5 (range, 76–90)	0.6668
Operation time (minute)	84.9 ± 9.2 (range, 63–101)	70.9 ± 10.2 (range, 58–90)	0.0004^a^
Blood Loss (ml)	145.9 ± 46.7 (range, 50–210)	125.3 ± 39.4 (range, 80–190)	0.1961
LLD (mm)	2.1 ± 1.9 (rang, 0–6)	8.8 ± 5.1 (range, 3–19)	<0.0001^a^

^a^Significant difference between CATMN group and Control group

## Discussion

The aim of hemiarthroplasty is to achieve normal function of the hip joint and equal limb length is a prerequisite for achieve normal function. However, limb-length discrepancy is a well-known complication of hip arthroplasty [8], and may cause uncomfortable problems for patients, such as limping or lower back pain [9].

Osteotomy and peroperative length measurement in hemiarthroplasty presents a challenge to orthopaedic surgeons. The accurate angle and height of osteotomy in hip arthroplasty is hard to control, which leads to limb discrepancy. Limb-length discrepancy (LLD) exceeding 10 mm after hip arthroplasty, raising legal or neurological issues, is reported in the literature [2, 10]. In order to achieve better control of the angle and height of osteotomy during hip arthorplasty, many surgeons developed various limb length measurement devices. Other studies have reported similar methods [6, 11–16] using pins, rulers, and calipers for intraoperative correction of limb-length discrepancy. For example, McGee et al. [14] reported a method using a wire placed in the ilium and stretched toward the greater trochanter. Woolson et al. [16] used a caliper fixed on the iliac wing, which obtained less than 6 mm LLD in 89% of cases. Some seem more effective than others, however, most studies considered only the limb-length control but not the angle and height of osteotomy, which plays an important role in leading to the LLD when the collared stem is used. The osteotomy of the femoral neck was usually performed 1 cm proximal to the lesser trochanter, directed 45° angle in the anteroposterior direction [17]. However, in clinical practice, the height and angle of osteotomy were depended on the experience of surgeon. In order to fill in the gap, we developed a device - ILMOD, to aid the osteotomy based on the preoperative measurement in X-ray film.

The concern over accuracy of the radiographic measurements may affect the measurement reliability. Measurements from X-ray image may further be subject to enlargement bias due to change in pelvis position with respect to the plane of the film and the centering of the X-rays [18]. In order to control this bias, we placed a long ruler with lead marks in the same frontal plane of femur for all patients when X-ray images were taken. Despite the efforts, on AP pelvic views, there may still be residual error because of X-ray divergence, as the rays are perpendicular not to the hip joint but to the midline of the pelvis.

In our study, although the operative time was prolonged in the ILMOD group, no wound infection including superficial infection was found. There are two possible reasons for this result. The first one is the time prolonged in ILMOD group is not very long, and the second one is the small sample size.

The limited choice of surgical approach, which ILMOD can be applied also poses a limitation on the device’s application. Due to the unique shape characteristics of the device, ILMOD can only be used in Kocher-Langenbeck approach. As part of ongoing efforts, we will try to develop more flexible devices that can be used in other approaches. And ILMOD is not suitable for dorsal comminuted fracture which cannot be anatomical reduced. In our study, all of 16 patients had performed anatomic reduction using more or less time. However, maybe a possible reason to explain all anatomic reconstruction of 16 patients is the small sample size. If no anatomic reconstruction of the femoral neck was performed, the usefulness of ILMOD would be reduced.

One other limitation is the short follow-up period. Despite the major complications (infection, fracture, dislocation and subsidence) were not observed in one month postoperatively, a long-term follow-up is necessary in the future to observe the real rate of complications.

## Conclusions

In summary, the present results show that the application of ILMOD can improve perioperative control of limb-length, and provides an accurate osteotomy plane during hemiarthroplasty. During operation, the ILMOD is fixed so that the measurement error due to the loosened device can be ignored. The ILMOD is easy to use and ensures an accurate osteotomy based on an accurate height measurement in hemiarthroplasty. The measurement can be used to aid the proper placement of the femoral component. There were only 31 patients enrolled in our study, a large sample prospective clinic trial is imperative to confirm the validity of ILMOD as an effective method to reduce LLD in hip arthroplasty.

## Abbreviations

ILMOD: Intraoperative limb-length measurement and osteotomy device
K-wire: Kirschner wire
LLD: Limb-length discrepancy
